# ASPS Exhibits Anti‐Rheumatic Effects by Reprogramming Gut Microbiota and Increasing Serumy‐Glutamylcysteine Leve

**DOI:** 10.1002/advs.202301728

**Published:** 2023-05-05

**Authors:** Ang Liu, Min Zhang, Yanglin Wu, Chenhui Zhang, Qin Zhang, Xinlin Su, Xu Zhu, Weidong Shi, Jiangyun Liu, Yang Zhang, Cheng Huang, Zhaowei Yan, Jun Lin

*Adv. Sci*. **2022**, *9*, 2205645

DOI: 10.1002/advs.202205645

Supporting Information file was replaced on May 5, 2023, after initial article online publication, to correct the description of the ‘immunofluorescence staining’ method.

## Supporting information

Supporting Information

